# The Effects Of Short Time Right Ventricular Pacing On Left Atrial Mechanical Functions

**Published:** 2009-05-15

**Authors:** Oyku Gulmez, Ilyas Atar, Elif Sade, I Asli Atar, Cagatay Ertan, Haldun Muderrisoglu, Bulent Ozin

**Affiliations:** Baskent University Faculty of Medicine, Department of Cardiology, Ankara

**Keywords:** Pacemaker, echocardiography, left atrial volume, left atrial function

## Abstract

**Objectives:**

Left atrium (LA) plays an important role in left ventricular filling. It is well known that right ventricular apical pacing has unfavorable effects on ventricular systolic and diastolic performance. The aim of this study is to evaluate the LA mechanical functions with 2D echocardiography in patients with a permanent pacemaker after short time ventricular pacing.

**Design:**

Echocardiographic examination was performed in 38 patients (mean age 63.0± 10.9, 18 female) with dual chamber pacemakers or defibrillators (< 20% ventricular pacing within previous 6 months, all of them on sinus rhythm) before and after 4 hours > 90% ventricular pacing at 70 beats per minute in DDD mode with an optimal AV interval. Left atrial volumes (LAV) including at the time of mitral valve opening (Vmax), at closure (Vmin), and at the onset of atrial systole (Volp) were measured. The passive emptying, conduit, active emptying and total emptying volume, stroke volumes were also calculated.

**Results:**

No significant differences were noted at baseline and after pacing for absolute Vmax, Volp, passive emptying, conduit, active emptying, total emptying volumes as well as the volumes indexed to body surface area (p >0.05).

**Conclusions:**

Short - time RV pacing seems to have no acute effects on left atrial mechanical functions.

## Introduction

The left atrium (LA) serves as a reservoir during ventricular systole, as a conduit during early diastole, and as an active contractile chamber that augment left ventricular filling in late diastole. Changes in LA and mechanic functions are associated with adverse clinical events such as atrial fibrillation, stroke, diastolic dysfunction, left ventricular failure [1-4]. Asynchronous ventricular activation induced by ventricular pacing can cause ventricular systolic and diastolic dysfunction and left ventricular dysfunction can be detected in 20-30% of patients with pacemakers [5,6]. Theoretically, abnormal LV filling induced by pacemakers should lead to LA enlargement and abnormal emptying. 

Echocardiography is the most commonly used non-invasive imaging technique for estimation of LA mechanical functions. The aim of the present study was to evaluate the acute effects of short time right ventricular AV sequentional pacing on LA mechanical functions based on 2D echocardiography.

## Materials and Methods

### Study Population

The study protocol was approved by the Baskent University Faculty of Medicine Ethics Committee. All patients provided written informed consent prior to participation in the study. The study population consisted of 38 patients with AV sequential pacemakers or defibrillators (ICD) with electrodes positioned in the RV apex and the right atrial appendage. Exclusion criteria included ≥ 20% ventricular pacing within the previous 6 months, atrial fibrillation (AF), atrial flutter, atrioventricular block, or pacemaker rhythm at the time of the study, previous hospitalization due to heart failure within the previous 3 months, ICD shocks within the previous month, significant valvular lesions, and poor imaging quality that precluded satisfactory echocardiographic assessment. All participants underwent conventional 2D echocardiography.

### Echocardiographic Study

Patients were imaged with an Acuson XP-256 (Acuson Siemens, Mountain View, California) machine equipped with a multi-frequency transducer in the left lateral decubitus position. The left atrial diameter was measured from the parasternal long axis, and the left atrial volumes were traced and calculated by means of a modified Simpson method from apical 4- and 2- chamber views according to the guidelines of the American College of Cardiology, American Heart Association, and American Society of Echocardiography [7]. Left ventricular end-diastolic and end-systolic volumes were determined from the apical 4- and 2- chamber views, and left ventricular stroke volume and ejection fraction EF were measured using the modified Simpson equation, with the use of apical views  [7]. Left atrial volumes were measured as: 1) just before mitral valve opening (maximal LA volume or Vmax); 2) at the onset of the P-wave on electrocardiography (pre-atrial contraction volume or Volp); and 3) at mitral valve closure (minimal LA volume or Vmin). From these, the following measurements were calculated:

1. LA passive emptying volume = Vmax-Volp;
2. LA passive emptying fraction = LA passive emptying volume/Vmax x 100;
3. LA conduit volume = LV stroke volume-(Vmax-Vmin);
4. LA active emptying volume = Volp-Vmin;
5. LA active emptying fraction = LA active emptying volume/Volp x 100;
6. LA total emptying volume = (Vmax-Vmin).
7. LA total emptying fraction = LA total emptying volume/Vmax x 100.

Atrial volumes were indexed to body surface area (BSA) in all patients.

After baseline echocardiographic evaluation, AV delay of the pacemakers was shortened at an optimal AV interval in which the paced rhythm at a heart rate of 70 beats per minute was achieved during the observation period of 5 minutes. After the observation period the patients left on paced rhythm during 4 h. After 4 h the pacemaker parameters were reprogrammed to their original settings and echocardiographic examinations were repeated. The percentage of ventricular paced beats was assessed by pacemaker telemetry. 

### Statistical Analyses

SPSS v.9.0 software (Statistical Package for the Social Sciences, SPSS Inc, Chicago, Ill, USA) was used for statistical analyses. Continuous variables with normal distribution were compared using the unpaired Student's t-test and are presented as mean ± SD. Continuous variables with abnormal distribution were compared using the Mann-Whitney U test. Echocardiographic measurements before and after short-time pacing were compared by means of the Wilcoxon signed-rank test. Categorical data were reported as frequencies and percentages. A p value < 0.05 was considered statistically significant.

## Results

The study population consisted of 38 (20 male, 18 female) patients, mean age 63.0 ± 10.9 years. Four (10.5%) patients had a pacemaker with VDD mode. The indications for pacemakers and/or ICDs were sick sinus syndrome in 22 (58.0%) patients, symptomatic sinus bradycardia in 1 (2.6%) patient, idiopathic ventricular tachycardia in 4 (10.5%) patients, ischemic ventricular tachycardia in 11 (28.9%) patients. Baseline atrial pacing was 11%, and ventricular pacing was 15%. Patient characteristics and echocardiographic data are listed in Table 1. Mild mitral regurgitation was present in 7 (18.7%) patients, and the degree of mitral regurgitation did not change after pacing. LA volume index more than 40 ml/m^2^  which is a criteria for severe LA dilatation according to The American Society of Echocardiography was present in 9 (23.7%) patients  [7,8]. 19 patients had an ejection fraction < 50%, and 12 had coronary artery disease. There were 15 (39.4%) patients with ICD, and the indication was idiopathic ventricular tachycardia in 4 (10.5%) of them. No rhythm disturbances were detected during the pacing period.

No significant difference was noted after short time pacing for absolute Vmax, Volp. Total emptying volume, passive emptying volume, active emptying volume, and conduit volume, as well as volumes indexed to BSA did not changed after short time pacing (all p> 0.05) (Table 2).

## Discussion

The purpose of the present study was to investigate the effects of short-time right ventricular apical pacing on LA mechanical functions based on 2D echocardiography. In this study, we have demonstrated that LA mechanical functions did not change significantly after 4-h right ventricular apical pacing.

Recent studies showed the unfavorable effects of asynchronous ventricular activation induced by ventricular pacing on ventricular functions  [9-11]. Data from the Mode Selection Trial and The Dual Chamber and VVI Implantable Defibrillator (DAVID) trial showed that increase in RV pacing resulted in increased risk of mortality and hospitalization for heart failure [10,12]. Moreover, right ventricular apical pacemakers had been shown to be the strongest independent predictor of LV EF decrease and worse cardiovascular outcomes over time [13].

Recent studies have shown that left atrial enlargement obtained from echocardiography is a good predictor of cardiovascular outcomes [1-3]. However, there are several limitations to estimate the LA size because of the irregular geometry of the LA. LA may enlarge asymmetrically, resulting in variable shape that may underestimate left atrial size, and it has been suggested that LA volume may be superior index to LA size [14,15]. The increase in LA volume is increasingly becoming a parameter of interest as a marker of overall cardiac function. To our knowledge, this is the first study to address the effects of short time pacing on LA mechanical functions. The results of our study suggest that > 90% AV sequential RV pacing for a short time did not have any negative effect on LA mechanical functions in the acute phase.

## Limitations

Our study has several limitations. First, LA volumes and mechanical functions were only investigated with 2D echocardiography. However, LA volume calculated by the biplane method has been shown to have a close correlation with that calculated by cine computed tomography, biplane angiography and with conventional 3D reconstruction which may not be feasible for routine clinical practice [16-18]. Second, the number of patients with different underlying cardiac diseases was relatively small in this study. As a consequence the sample size was not sufficient to make subgroup analysis.

## Conclusion

Short time AV sequentional RV apical pacing seems to have no acute effects on LA mechanical functions when evaluated with on 2D echocardiographic evaluation. However, large and prospective studies, and other invasive or noninvasive methods such as tissue Doppler, strain, strain rate, hemodynamic measurements to evaluate atrial mechanical functions are required to confirm our findings.

## Figures and Tables

**Table 1:**
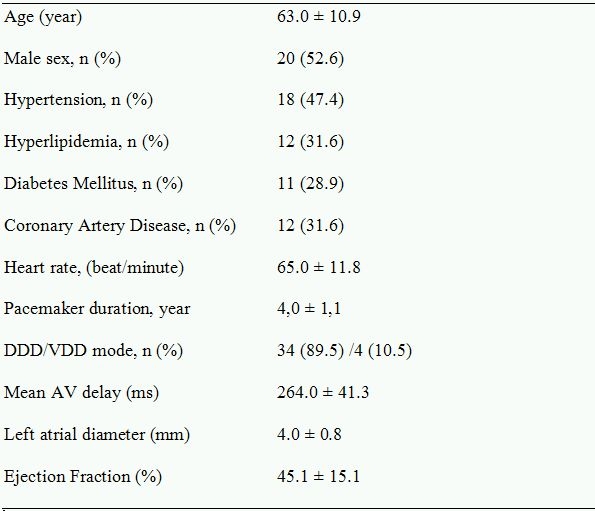
Demographic and Echocardiographic Variables of the Study Population

**Table 2:**
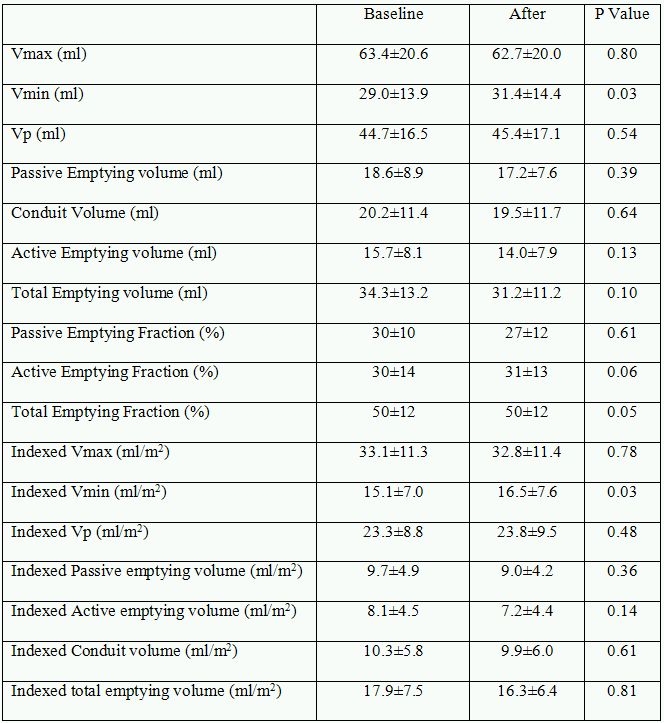
Comparison of left atrial volumes and fractions before and after short-time pacing
